# Cardiac calcified amorphous tumor in a patient with lung cancer

**DOI:** 10.1186/s44215-024-00161-7

**Published:** 2024-08-13

**Authors:** Kyohei Hatori, Jun Mohara, Satoru Shibata, Miyuki Murata, Nobuaki Fukuda, Shitoshi Hiroi, Tetsuya Koyano

**Affiliations:** 1 Department of Cardiovascular Surgery, NHO Takasaki General Medical Center, Gunma, Japan; 2 Department of Cardiology, NHO Takasaki General Medical Center, Gunma, Japan

**Keywords:** Calcified amorphous tumor, Lung cancer, Cancer-related thrombosis, Non-bacterial thrombotic endocarditis

## Abstract

**Background:**

Calcified amorphous tumor of the heart is a rare non-neoplastic cardiac mass composed of calcified nodules over amorphous fibrous tissue with degeneration and some chronic inflammation. Calcified amorphous tumor is often associated with mitral annular calcification in patients with end-stage renal disease on dialysis. However, the exact etiology of calcified amorphous tumors remains uncertain.

**Case presentation:**

A 77-year-old female with lung cancer showed a tumor with large mobility in the left ventricular outflow tract on transthoracic echocardiography. She had mitral annular calcification, although her renal function was normal. The tumor was excised surgically. Pathologically, the extracted specimen consisted of a calcified lesion without tumor tissue and was diagnosed as a calcified amorphous tumor.

**Conclusions:**

As the patient had no other risk factors for calcified amorphous tumor except mitral annular calcification, we considered the association of blood coagulation abnormalities due to cancer-related thrombosis. This case suggests that calcified amorphous tumors may be associated with malignant tumors.

## Background

Calcified amorphous tumor (CAT) of the heart is a rare non-neoplastic cardiac mass composed of calcified nodules within amorphous fibrous tissue [1]. Although the exact etiology of CAT remains uncertain, it is often associated with mitral annular calcification (MAC) in patients with end-stage renal disease on dialysis [2]. Other risk factors include blood coagulation abnormalities, such as diabetes or hematological disorders [3]. Interestingly, patients with cancer are also susceptible to blood coagulation abnormalities, which contribute to cancer-related thrombosis [4]. In patients with malignant tumors, hypercoagulability and metabolic abnormalities are often present. Despite these being considered risk factors for CAT, reports of CAT in patients with malignant tumors are scarce. This report describes a case of CAT with normal renal function suspected to be associated with lung cancer.

## Case presentation

**Fig. 1:**
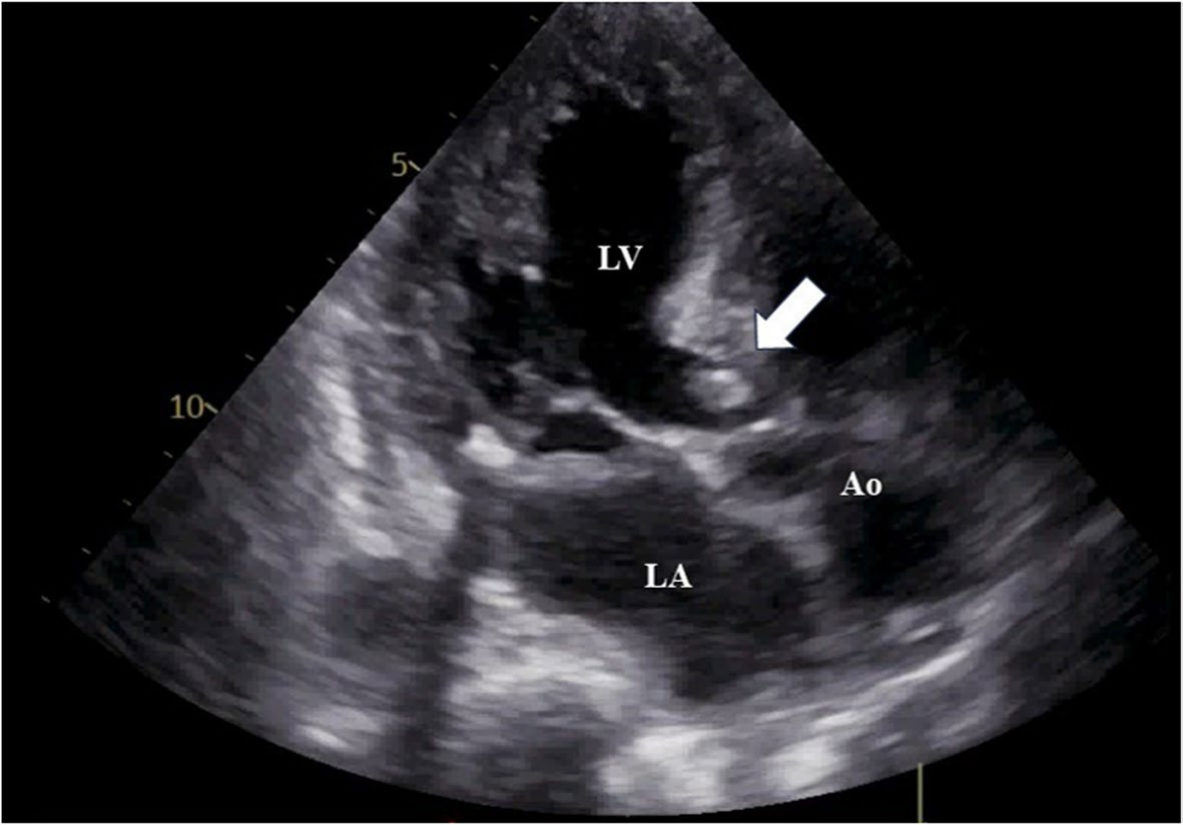
Preoperative transthoracic echocardiography shows a tumor with large mobility(arrow) in the left ventricular outflow tract. LV: left ventricle, LA: left atrium, Ao: aorta

**Fig. 2:**
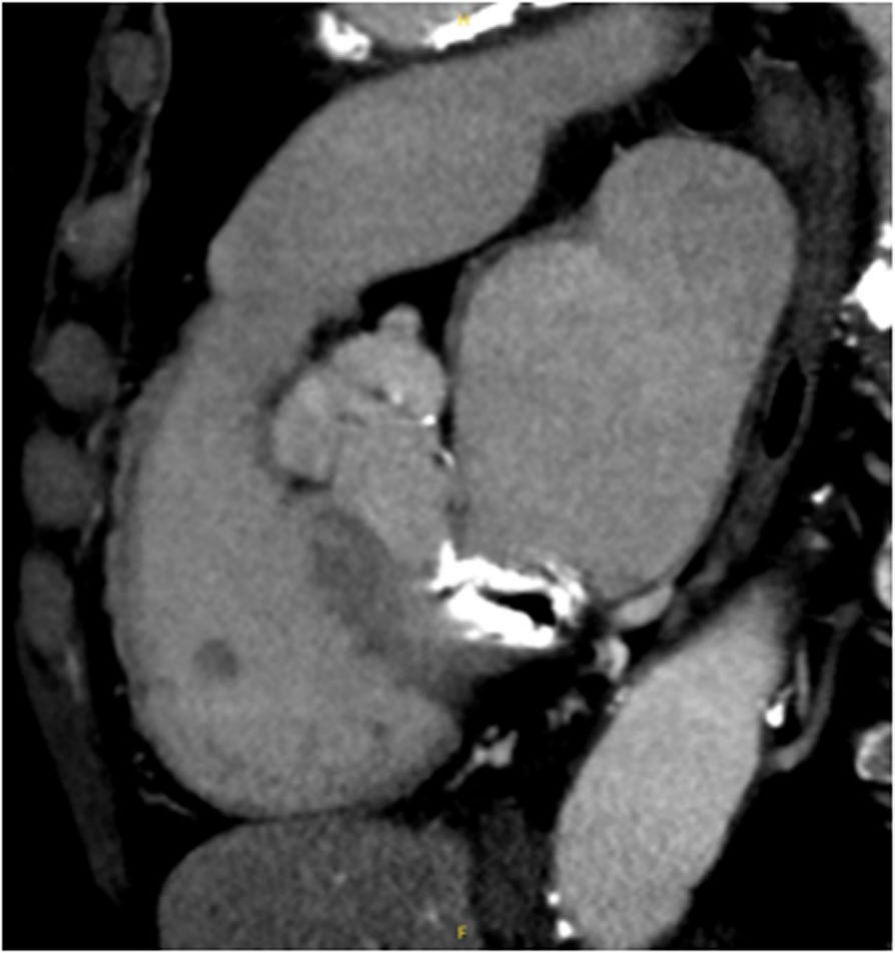
Preoperative computed tomography shows MAC in the mitral valve. MAC: mitral annular calcification

**Fig. 3:**
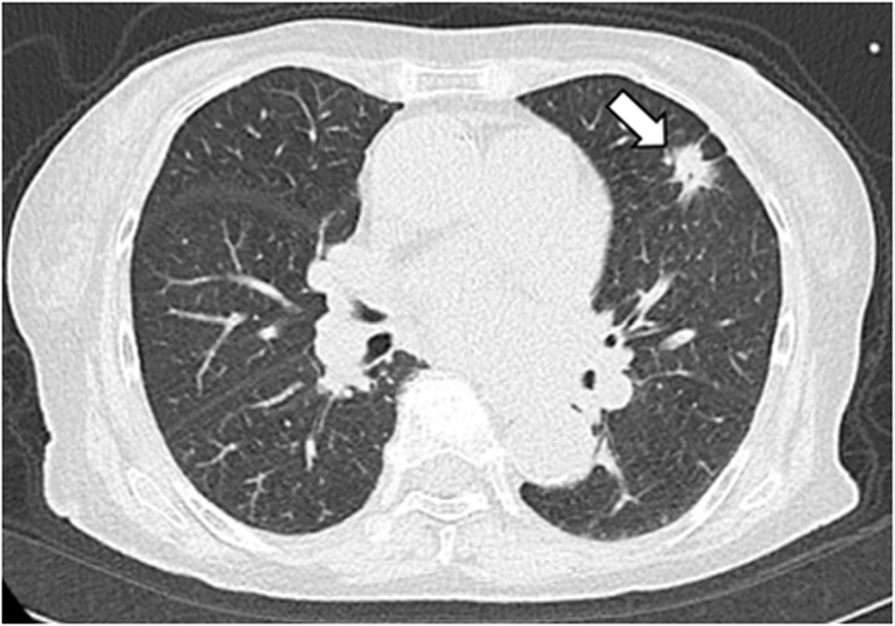
Preoperative computed tomography shows a nodular shadow of 1.4 cm (arrow) under the pleura of the S3 area of the left lung

**Fig. 4:**
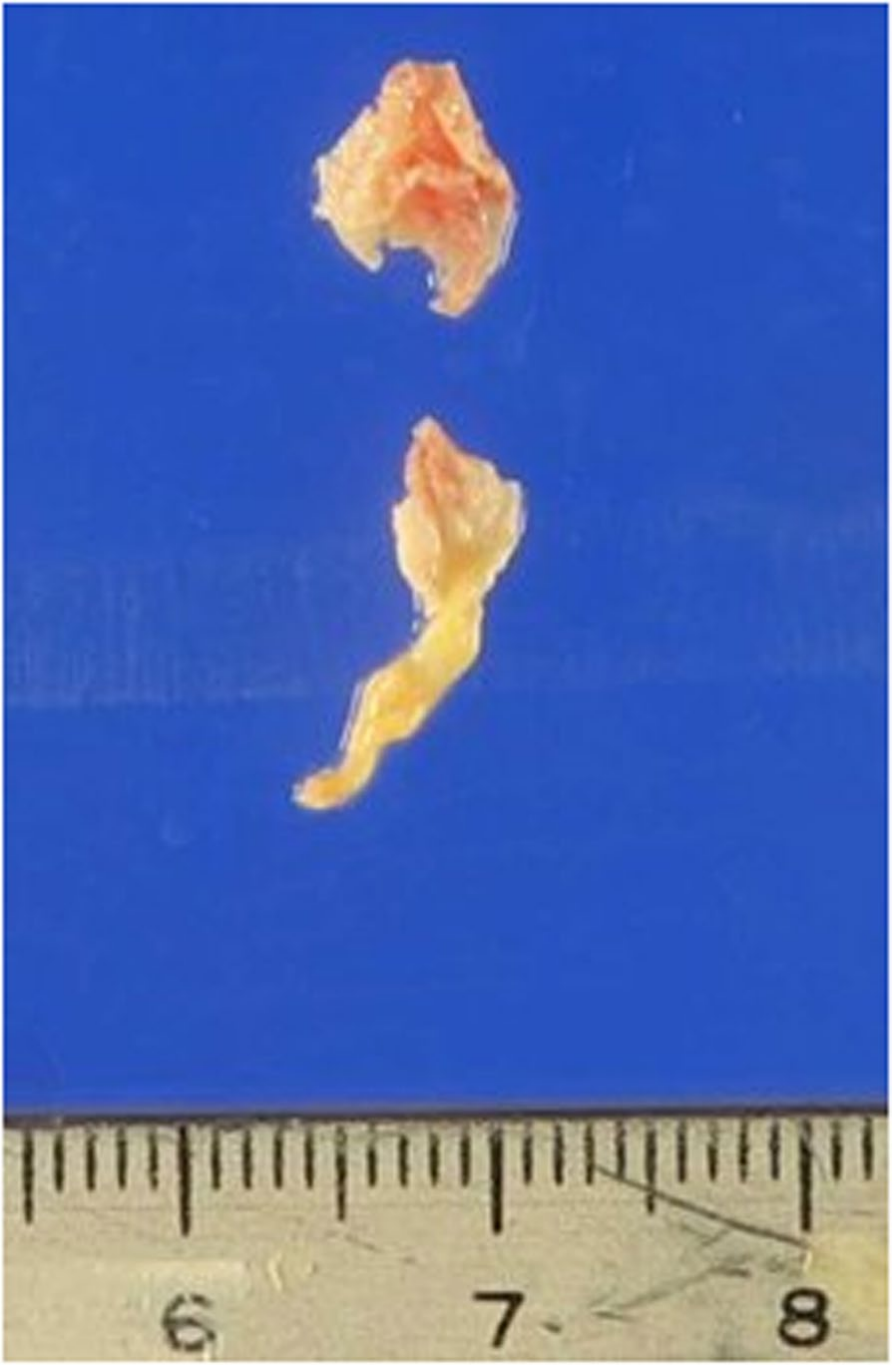
Photograph of the excised tumor. A milky-white, small-finger-sized pedunculated tumor was excised

**Fig. 5:**
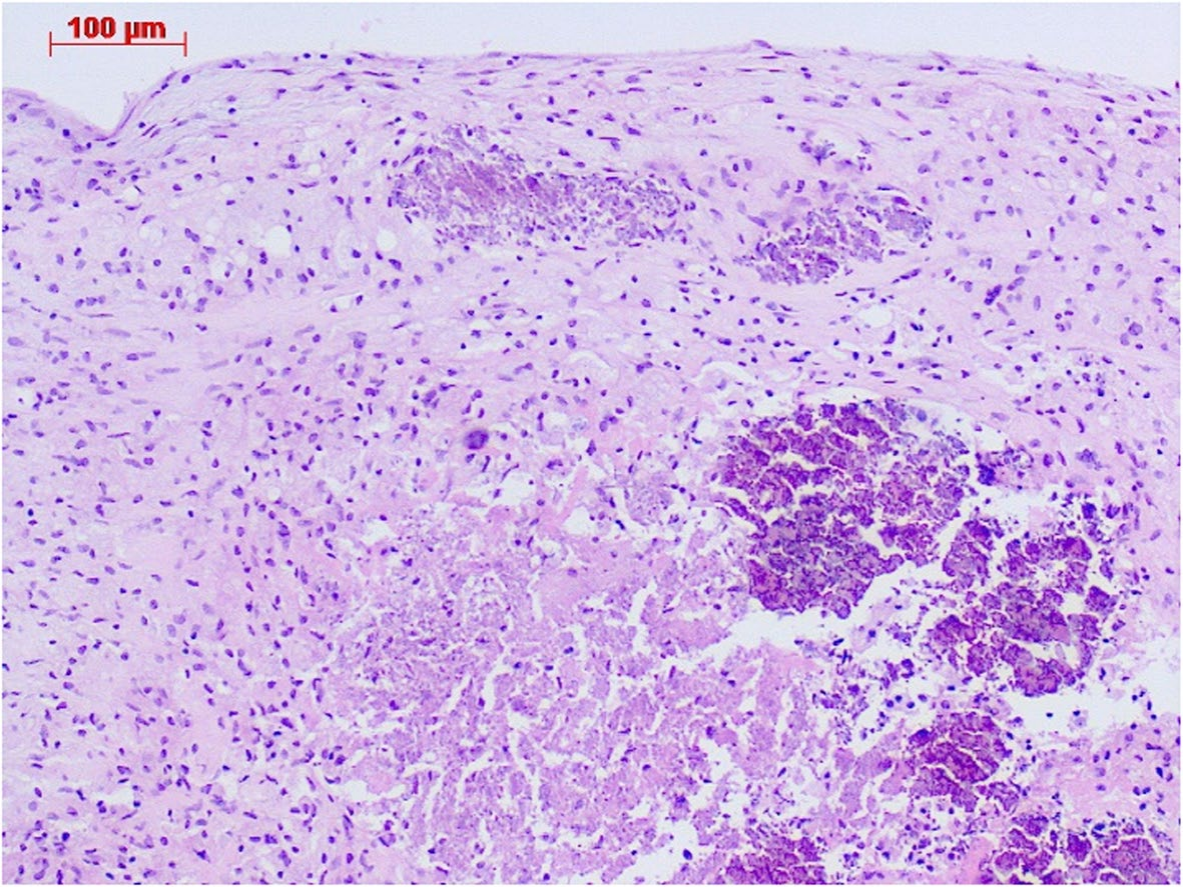
Pathological examination shows numerous island-like calcified nodules including fibrin, chronic inflammation, and necrotic substances, confirming the diagnosis of a calcified amorphous tumor. Hematoxylin and eosin stain

A 77-year-old woman with a history of hypertension was admitted with a chief complaint of dyspnea and chest pain. Hematological and biochemical tests showed no signs of infection, with a white blood cell count of 7000/μl and a C-reactive protein level of 0.35 mg/dl. In the blood culture test, no bacteria were detected. Her creatinine level was 0.85 mg/dl, and she did not have renal failure. She was not diabetic with an HbA1c level of 6.1%. Her calcium metabolism was normal, with a calcium level of 9.1 mg/dl. Her preoperative D-dimer was slightly elevated at 1.30 μg/ml. A 12-lead electrocardiogram showed sinus rhythm and negative T waves in the anterior chest leads. Transthoracic echocardiography (TTE) showed normal left ventricular systolic function (left ventricular ejection fraction 60%), mild calcification of the aortic valve, and severe calcification of the mitral annulus. TTE also revealed a tumor with significant mobility in the left ventricular outflow tract attached to the non-coronary cusp and the right coronary cusp (Fig. 1). No thrombus was observed in the left atrium. Computed tomography (CT) examination demonstrated MAC in the mitral valve (Fig. 2). In addition, CT revealed a nodular shadow measuring 1.4 cm in the S3 area of the left lung (Fig. 3). No lymph node enlargement was observed in the hilum or mediastinum. Given these findings, primary lung cancer was suspected. Furthermore, no thrombus was observed in the arteries and veins. The cardiac catheterization revealed 90% stenosis in segment #2 of the right coronary artery. Differential diagnosis of a tumor-like lesion in the left ventricle involved thrombus, primary cardiac tumor, metastatic tumor, or vegetation. Because of the high mobility of the tumor, the risk of embolism was considered significant. Tumor resection and coronary artery bypass grafting were deemed appropriate. After discussions with the respiratory team, it was determined that a detailed examination of lung cancer would be difficult in situations with a high risk of embolism from cardiac tumorous lesions. Therefore, the decision was made to prioritize heart surgery. The surgery was approached with a median sternotomy, and the great saphenous vein was harvested from the lower leg. Cardiopulmonary bypass was established with aortic and right atrial cannulations, and cardiac arrest was induced with antegrade cold-blood cardioplegia. A transverse incision was made in the ascending aorta, revealing a mobile, milky-white, thumb-sized pedunculated tumor attached just below the junction of the right and non-coronary cusps near the membranous septum (Fig. 4). It was sharply excised from the endocardial attachment site. No thrombus was observed in the left ventricular cavity. After the closure of the aortic incision, coronary artery bypass grafting was performed. Pathological examination revealed that the extracted lesion was composed of numerous island-like calcified nodules on a background of thrombus-like fibrinoid material deposition, fibrous granulation tissue, and nest-like degenerative and necrotic substances (Fig. 5). No malignant findings were observed. Then, a diagnosis of CAT was obtained. The postoperative course was good, and the patient was discharged from the 22 hospital days after the surgery. Although we had scheduled examinations and treatment for lung cancer, we failed to proceed because of the rapid deterioration of the patient’s condition. CT scans taken 3 months after the surgery revealed mediastinal lymph node metastasis, adrenal metastasis, and tracheal invasion of the lung tumor. Blood tests revealed an elevated level of pro-gastrin-releasing peptide at 89.3 pg/ml, leading to a clinical diagnosis of small-cell lung cancer. Because of bleeding from the airway, antiplatelet drugs were discontinued. A CT scan taken 4 months after the surgery revealed a thrombus in the left atrium. The patient’s cardiac rhythm was sinus; therefore, it was considered tumor-related thrombosis. There was no recurrence of the left ventricular outflow tract CAT. The tumor’s tracheal invasion progressed further, and the patient died 5 months after the surgery.

## Discussion

CAT is a rare non-neoplastic intracardiac mass composed of calcium deposition and amorphous fibrinous materials. It was first described in 1997 by Reynolds [1]. CAT usually appears as a calcified intracardiac tumor that may be located in any cardiac chamber, and its size can vary from small punctate lesions to numerous masses. Pathophysiological hypotheses involving an organized thrombus origin favored by hypercoagulability and/or phosphocalcium metabolism abnormalities have been proposed. Patients with CAT frequently present with concomitant conditions such as pre-existing valve disease (MAC for instance), end-stage renal disease, or other cardiovascular risk factors [5]. Other risk factors include blood coagulation abnormalities, such as diabetes or hematological disorders [3].

Malignant tumors are a representative disease that causes abnormal blood coagulation and chronic inflammation. The condition is known as cancer-related thrombosis [4]. In patients with malignancy, the interaction between macrophages, monocytes, and malignant cells releases interleukins, tumor necrosis factor, and tissue factor, causing endothelial damage, which provides a surface for the initiation of platelet aggregation and subsequent hypercoagulability and thrombogenesis [6]. It is reported that 9.8% of lung cancer patients experienced cancer-related thrombosis [7]. Small cell lung cancer, as seen in this case, is particularly prone to thromboembolic events.

Non-bacterial thrombotic endocarditis (NBTE) is a tumor-like lesion within the heart, sharing similarities with CAT. NBTE is a disease characterized by the presence of vegetations, which consist of fibrin and platelet aggregates and are devoid of inflammation or bacteria. NBTE is known to occur in various pathologies that cause endothelial damage such as malignant tumors, autoimmune diseases (rheumatoid arthritis, systemic lupus erythematosus, antiphospholipid antibody syndrome), chronic wasting diseases (acquired immune deficiency syndrome, uremia, tuberculosis), coagulation abnormalities, sepsis, radiation therapy or overdose [8].

While there are numerous reported cases of NBTE associated with cancer [6, 8–10], relatively few cases of CAT have been documented in cancer patients [11, 12]. Furthermore, no literature has described the relationship between NBTE and CAT. NBTE is believed to occur in areas of cardiac endothelial damage caused by turbulence and regurgitation. Considering the patient’s concurrent presence of malignant tumors, it was thought that endothelial damage was the underlying cause. This may lead to the formation of NBTE-like lesions in the left ventricular outflow tract. The persistence of inflammation and a hypercoagulable state may ultimately result in CAT. We cannot rule out the possibility that CAT has only coincidentally occurred in patients with lung cancer. Unfortunately, CAT itself is a rare condition with few reported cases, and its mechanism remains unclear. Alizadehasl et al. have reported cases of CAT in patients with colorectal cancer [11], while Hussain et al. have reported CAT cases in patients with adenocarcinoma of the lung [12]. In patients with malignant tumors, their overall condition is often poor, which may result in a reduced likelihood of undergoing heart surgery. Consequently, there is a possibility that no CAT diagnosis has been pursued. Further accumulation of cases is considered necessary to explain the relationship between CAT and malignant tumors.

## Conclusion

We report a case in which surgical treatment was performed for a mobile tumor that had formed in the left ventricular outflow tract. The diagnosis of CAT was made on the basis of pathological findings. The patient had no risk factors for CAT other than MAC, so it was suspected to be associated with lung cancer. Because the cause of CAT remains unclear, further investigation has proven challenging. We hope that the accumulation of additional reported cases and knowledge will lead to the elucidation of its underlying cause.

## Data Availability

Not applicable.

## Abbreviations

CAT: Calcified amorphous tumor
MAC: Mitral annular calcification
TTE: Transthoracic echocardiography
CT: Computed tomography
NBTE: Non-bacterial thrombotic endocarditis
